# Care pathway for autistic children and their families in Europe

**DOI:** 10.1192/j.eurpsy.2022.1119

**Published:** 2022-09-01

**Authors:** M.A. Mendez, A. San Jose Caceres, B. Oakley, D. Murphy, C. Arango, M. Parellada, R. Canitano, V. Quoidbach

**Affiliations:** 1 Hospital Universitario Gregorio Marañon, Instituto De Investigación Sanitaria, Madrid, Spain; 2 King’s College London, Forensic And Neurodevelopmental Sciences, AF, United Kingdom; 3 Hospital Gregorio Marañon, Department Of Child And Adolescent Psychiatry,, Madrid, Spain; 4 General University Hospital of Siena, Division Of Child And Adolescent Neuropsychiatry, Siena, Italy; 5 European Brain Council, Public Health & Policy Department, Brussels, Belgium

**Keywords:** Autism, Early screening/diagnosis, early intervention, policy recommendations

## Abstract

**Introduction:**

Autism is a lifelong complex neurodevelopmental condition that affects brain development and behaviour with significant consequences for everyday life (WHO, 2018). Despite its personal, familial and societal impact, there is still a European-wide lack of harmonised guidelines about the support needed from early stages, the most sensitive time to gain positive future outcomes (Berajamo-Martin et al, 2019).

**Objectives:**

The objectives were: 1. To analyse autistic children care pathway and patient/carer journey in three European countries: Italy, Spain and U.K. 2. To propose policy recommendations on how to improve this pathway.

**Methods:**

To identify major barriers and treatment gaps, we conducted a rapid literature review of the care pathway in Europe and a survey aimed at parents or carers of autistic children ages 0 to 18 living in the three countries. The survey gathered information on screening, diagnosis, accessibility and support received before, during and after diagnosis. Members of the working group met to discuss results and propose policy recommendations.

**Results:**

1. Current care pathway analysis showed the following treatment gaps: Long waiting time from first concerns until screening visit and confirmed diagnosis. Delayed or no access to intervention once diagnosis has been confirmed. Overall limited information about autism and how to access early detection services. Overall deficient support to families. 2. Please see Box 1 for our proposed policy recommendations.

**Box 1. Policy recommendations**

**Figure:**
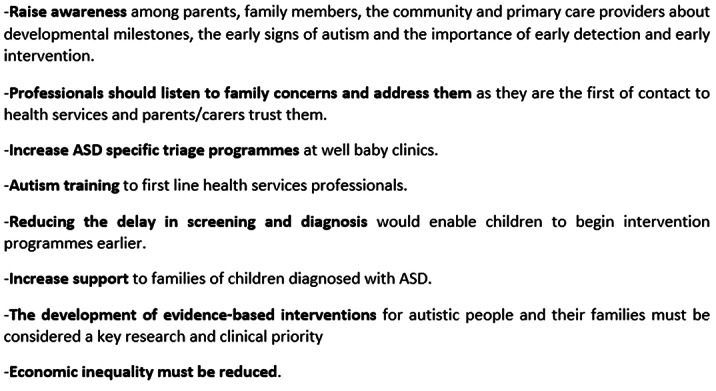

**Conclusions:**

Our findings and recommendations will inform policy harmonisation in Europe to shorten long waiting times, diagnosis process and intervention, and therefore, improve autistic people and their families’ journey experience and quality of life.

**Disclosure:**

No significant relationships.

